# Supplementary material for: Associations of Sex Steroids and Sex Hormone-Binding Globulin with Non-Alcoholic Fatty Liver Disease: A Population-Based Study and Meta-Analysis
Source: Genes (Basel). 2022 May 27;13(6):966. doi: 10.3390/genes13060966 (PMC9223113; doi:10.3390/genes13060966)
Supplement: Supplementary file 1 [file genes-13-00966-s001.zip › genes-1713965-supplementary.pdf]

Supplementary Methods S1

Assessment of covariates in the Rotterdam Study

Information on current health status, smoking behavior, medication use, and menopausal status, was obtained during home interviews. Height and weight were measured, and the body mass index (BMI) [(weight in kg)/(height in m)^2] was calculated. Smoking status was categorized into current, former, or never smokers. Further smoking status was categorized into two groups including never-smokers and ever-smokers (current and former smokers combined). Alcohol consumption was assessed in grams of ethanol per day. Hypertension was defined as systolic blood pressure (BP) ≥140 mm Hg or a diastolic BP ≥90 mm Hg or the use of BP-lowering drugs prescribed for hypertension. T2D was defined according to recent WHO guidelines. Participants who fulfilled at least one of the following criteria were considered as having incident T2D i) fasting blood glucose ≥7.0 mmol/L ii) non-fasting blood glucose between ≥11.1 mmol/L iii) the use of anti-diabetic medication. Hepatitis B surface antigen, and anti-hepatitis C virus were measured by an automatic immunoassay (Roche Diagnostics GmbH). High-density lipoprotein (HDL) cholesterol, total cholesterol, and triglycerides were measured on the COBAS 8000 Modular Analyzer (Roche Diagnostics GmbH). We assessed medication use for hypertension, hyperlipidemia, and diabetes through interview data. Current hormone use was defined as use of hormone medication within 3 months before the date of blood collection. Medication with Anatomical Therapeutic Chemical code g03 sex hormones and modulators of the genital system were included. Postmenopausal women were defined as women who reported the absence of a menstrual cycle for at least one year. Age at menopause was defined as self-reported age at the time of last menstruation.

Statistical analysis in the Rotterdam Study

Continuous variables were expressed as mean (standard deviation) or median (25th percentile–75th percentile), and categorical variables were presented as sample sizes and percentages. Because the missing values were likely to be missing at random and for avoidance of loss in efficiency, missing values on covariates (ranging from 0.1% to 5.0%) were imputed using a multiple imputation technique (N =5 imputations). All analyses were performed using SPSS

statistical software (SPSS, version 25; IBM Corp) and R software version 4.0.2 (The R Foundation for Statistical Computing).

All analyses were stratified by sex. Multivariable logistic regression models were used to evaluate whether sex steroids and SHBG were associated with non-alcoholic fatty liver disease (NAFLD). All sex hormone variables were assessed in separate models, continuously and in tertiles. For total estradiol, the first tertile included all women with levels of total estradiol lower than the detection limit. To achieve approximately normal distribution, skewed variables (alcohol consumption, cholesterol, triglycerides, total estradiol, total testosterone, SHBG, DHEA, DHEAS) were naturally log transformed. Odds ratio (OR), 95% confidence intervals (95%CI) and P trend were reported. A p-value lower than 0.05 was considered as statistically significant. In a more conservative approach and to take into account multiple testing, we applied a conservative Bonferroni corrected p-value <0.01 (0.05/5) for 5 tests (total estradiol, total testosterone, SHBG, DHEA and DHEAS). In the basic model (model 1), we adjusted the analysis for age, time difference between hormone measurements and performed abdominal ultrasounds, BMI, ever smoking (yes vs no) and alcohol consumption. In model 2, potential risk factors and confounders of NAFLD were added to model 1 including hypertension (yes vs no), type 2 diabetes mellitus (yes vs no), HDL, triglycerides, and menopausal status (only in women). We also conducted similar analysis to investigate the association of sex hormones with MAFLD in model 2.

For sensitivity analyses, we additionally adjustment for waist circumference, using hormone medication, serum lipid lowering medication or sex hormones for each other to model 2. Moreover, we performed stratified analysis by BMI (<25, 25-30 and ≥30) or age (<50 years, 50-60 years and ≥60 years) groups to explore if the associations were similar with the BMI or age categories. We repeated the analyses among postmenopausal women, and we repeated the analyses stratifying by time since menopause (<5, 5-10 and ≥10) among postmenopausal women to see if the association was similar across the categories of time since menopause.

Supplementary Table S1. Sensitivity analysis of sex steroids and SHBG with NAFLD among men.

	Total estradiol	Total testosterone	SHBG	DHEA	DHEAS
Multivariable model 2	0.87 (0.72-1.06)	0.92 (0.74-1.13)	**0.72 (0.59-0.88)****	0.91 (0.79-1.04)	0.98 (0.91-1.06)
Multivariable model 2 + waist circumference	0.87 (0.71-1.06)	0.93 (0.76-1.15)	**0.72 (0.59-0.88)****	0.92 (0.81-1.05)	1.01 (0.88-1.18)
Multivariable model 2+ use of hormone medication	0.87 (0.72-1.06)	0.92 (0.74-1.13)	**0.72 (0.59-0.88)****	0.90 (0.79-1.03)	1.00 (0.87-1.16)
Multivariable model 2+ lipid lowering medication	0.87 (0.72-1.06)	0.92 (0.75-1.14)	**0.72 (0.59-0.88)****	0.91 (0.79-1.04)	1.00 (0.87-1.16)
Multivariable model 2 + sex hormones for each other	0.90 (0.73-1.12)	1.12 (0.92-1.59)	**0.67 (0.51-0.89)****	0.84 (0.71-0.98)	1.02 (0.87-1.21)
BMI (kg/m^2) [a]					
<25 (n=185)	0.76 (0.57-1.01)	**0.55 (0.37-0.80)****	**0.60 (0.44-0.82)****	0.91 (0.74-1.13)	0.97 (0.76-1.23)
25-30 (n=409)	0.88 (0.65-1.20)	1.03 (0.78-1.38)	0.73 (0.53-0.99)*	0.89 (0.73-1.08)	0.97 (0.79-1.20)
≥ 30 (n=161)	1.00 (0.65-1.55)	0.91 (0.54-1.55)	0.86 (0.56-1.32)	0.97 (0.71-1.32)	1.11 (0.80-1.53)
Age (years) [a]					
<50 (n=114)	0.97 (0.61-1.56)	1.57 (0.81-3.04)	1.11 (0.68-1.83)	0.83 (0.59-1.16)	1.16 (0.79-1.70)
50-60 (n=446)	0.84 (0.65-1.08)	0.94 (0.73-1.22)	**0.68 (0.51-0.89)****	0.94 (0.78-1.13)	0.93 (0.76-1.14)
≥ 60 (n=195)	0.78 (0.48-1.27)	0.62 (0.39-1.00)*	**0.60 (0.41-0.88)****	0.87 (0.68-1.10)	1.00 (0.78-1.29)

The p-values surpassing the significance threshold (p-value< 0.05). * p-value<0.05, ** p-value<0.01. Association remains significant at a Bonferroni corrected p-value <0.01 for 5 tests are bold. [a] Results are adjusted for variables in model 2. Model 2: age+ time difference between hormone measurement and performed ultrasound+ BMI+ ever smoking + alcohol consumption+ hypertension+T2D+ HDL+ triglycerides. Abbreviations: NAFLD, non-alcoholic fatty liver disease; SHBG, sex hormone-binding globulin; DHEA, Dehydroepiandrosterone; DHEAS, Dehydroepiandrosterone sulfate; BMI, body mass index; T2D, type 2 diabetes; HDL, high density lipid.

Supplementary Table S2. Sensitivity analysis of sex steroids and SHBG with NAFLD among women.

	Total estradiol	Total testosterone	SHBG	DHEA	DHEAS
**Women**					
Multivariable model 2	1.01 (0.96-1.06)	0.85 (0.76-0.96)*	**0.69 (0.61-0.78)*** **	1.01 (0.92-1.11)	1.01 (0.92-1.10)
Multivariable model 2+ waist circumference	1.01 (0.96-1.06)	0.87 (0.78-0.99)*	**0.71 (0.63-0.80)*** **	1.00 (0.92-1.10)	1.00 (0.92-1.09)
Multivariable model 2+use of hormone medication	1.01 (0.96-1.06)	**0.85 (0.75-0.96)** **	**0.67 (0.59-0.76)*** **	1.00 (0.92-1.10)	1.01 (0.92-1.10)
Multivariable model 2+ lipid lowering medication	1.01 (0.96-1.06)	0.86 (0.76-0.96)*	**0.69 (0.61-0.78)*** **	1.00 (0.92-1.10)	1.01 (0.92-1.10)
Multivariable model 2 + sex hormones for each other	1.03 (0.98-1.09)	0.87 (0.76-1.00)*	**0.69 (0.61-0.78)*** **	1.08 (0.96-1.22)	1.01 (0.91-1.12)
BMI (kg/m^2) [a]					
<25 (n=362)	1.02 (0.96-1.08)	1.02 (0.88-1.19)	0.99 (0.84-1.17)	1.01 (0.90-1.13)	0.99 (0.89-1.11)
25-30 (n=440)	1.02 (0.94-1.11)	0.86 (0.69-1.07)	**0.62 (0.50-0.76)*** **	1.01 (0.86-1.18)	1.15 (0.99-1.35)
≥ 30 (n=307)	0.93 (0.81-1.06)	**0.67 (0.51-0.87)** **	**0.56 (0.44-0.73)*** **	1.00 (0.81-1.23)	0.89 (0.72-1.08)
Age (years) [a]					
<50 (n=163)	1.00 (0.91-1.10)	0.81 (0.58-1.14)	0.88 (0.68-1.15)	0.91 (0.72-1.15)	0.90 (0.71-1.15)
50-60 (n=666)	1.01 (0.94-1.08)	0.92 (0.79-1.08)	**0.72 (0.62-0.84)*** **	1.03 (0.92-1.16)	1.02 (0.91-1.14)
≥ 60 (n=280)	0.93 (0.75-1.15)	0.70 (0.54-0.90)	**0.47 (0.34-0.64)*** **	0.98 (0.81-1.18)	1.02 (0.85-1.23)
**Postmenopausal women**					
Multivariable model 2	0.99 (0.90-1.10)	**0.80 (0.68-0.94)** **	**0.62 (0.51-0.75)*** **	1.05 (0.93-1.20)	1.02 (0.90-1.16)
Time since menopause (years) [a]					
<5 (n=174)	1.08 (0.91-1.27)	1.08 (0.78-1.51)	**0.57 (0.40-0.82)** **	1.11 (0.85-1.46)	1.11 (0.86-1.42)
5-10 (n=199)	1.06 (0.86-1.31)	0.80 (0.60-1.06)	0.74 (0.50-1.08)	1.09 (0.90-1.32)	1.04 (0.86-1.27)
≥10 (n=209)	0.88 (0.75-1.04)	**0.68 (0.51-0.89)** **	**0.58 (0.43-0.78)*** **	1.01 (0.79-1.28)	0.93 (0.73-1.18)

The p-values surpassing the significance threshold (p-value< 0.05). * p-value<0.05, ** p-value<0.01, ***p-value<0.001. Association remains significant at a Bonferroni corrected P value <0.01 for 5 tests are indicated in bold. [a] Results are adjusted for variables in model 2. Model 2: age+ time difference between hormone measurement and performed ultrasound+ BMI+ ever smoking + alcohol consumption+ hypertension+T2D+ HDL+ triglycerides+ postmenopausal status. Abbreviations: NAFLD, non-alcoholic fatty liver disease; SHBG, sex hormone-binding globulin; DHEA, Dehydroepiandrosterone; DHEAS, Dehydroepiandrosterone sulfate; BMI, body mass index; T2D, type 2 diabetes; HDL, high density lipid.

Supplementary Table S3. Associations between sex steroids and SHBG with MAFLD in model 2.

	Total estradiol	Total testosterone	SHBG	DHEA	DHEAS
**Men**					
Tertile 1, OR (95%CI)	1 (Reference)	1 (Reference)	1 (Reference)	1 (Reference)	1 (Reference)
Tertile 2, OR (95%CI)	1.24 (0.81-1.91)	0.86 (0.57-1.32)	0.67 (0.44-1.02)	1.02 (0.66-1.59)	1.11 (0.71-1.72)
Tertile 3, OR (95%CI)	0.81 (0.52-1.27)	0.78 (0.50-1.23)	**0.50 (0.32-0.78)****	0.76 (0.48-1.19)	1.32 (0.84-2.08)
Continuous	0.88 (0.73-1.06)	0.96 (0.78-1.17)	**0.75 (0.62-0.91)****	0.93 (0.82-1.06)	1.08 (0.91-1.27)
P trend	0.18	0.68	0.004	0.28	0.38
**Women**					
Tertile 1, OR (95%CI)	1 (Reference)	1 (Reference)	1 (Reference)	1 (Reference)	1 (Reference)
Tertile 2, OR (95%CI)	1.14 (0.76-1.70)	0.98 (0.67-1.43)	**0.49 (0.34-0.71)*****	1.14 (0.78-1.67)	1.10 (0.74-1.61)
Tertile 3, OR (95%CI)	1.09 (0.73-1.63)	0.78 (0.52-1.16)	**0.29 (0.18-0.44)*****	1.06 (0.71-1.57)	1.00 (0.67-1.49)
Continuous	1.00 (0.95-1.05)	**0.85 (0.75-0.95)****	**0.68 (0.61-0.77)*****	1.00 (0.92-1.09)	1.00 (0.93-1.10)
P trend	0.95	0.006	1.69×10^{-10}	0.99	0.83

The p-values surpassing the significance threshold (p-value<0.05). ** p-value<0.01, *** p-value<0.001. Association remains significant at a Bonferroni corrected P value <0.01 for 5 tests are indicated in bold. Model 2: age+ time difference between hormone measurement and performed ultrasound+ BMI+ ever smoking + alcohol consumption+ hypertension+T2D+ HDL+ triglycerides + postmenopausal status (only in women). Abbreviations: MAFLD, metabolic dysfunction-associated fatty liver disease; SHBG, sex hormone-binding globulin; DHEA, Dehydroepiandrosterone; DHEAS, Dehydroepiandrosterone sulfate; BMI, body mass index; T2D, type 2 diabetes; HDL, high density lipid. SHBG, sex hormone-binding globulin; DHEA, Dehydroepiandrosterone; DHEAS, Dehydroepiandrosterone sulfate; BMI, body mass index; T2D, type 2 diabetes; HDL, high density lipid.

Supplementary Table S4. PRISMA checklist

Section/topic	#	Checklist item	Reported on page #
**TITLE**			
Title	1	Identify the report as a systematic review, meta-analysis, or both.	1
**ABSTRACT**			
Structured summary	2	Provide a structured summary including, as applicable: background; objectives; data sources; study eligibility criteria, participants, and interventions; study appraisal and synthesis methods; results; limitations; conclusions and implications of key findings; systematic review registration number.	1
INTRODUCTION			
Rationale	3	Describe the rationale for the review in the context of what is already known.	2
Objectives	4	Provide an explicit statement of questions being addressed with reference to participants, interventions, comparisons, outcomes, and study design (PICOS).	2
**METHODS**			
Protocol and registration	5	Indicate if a review protocol exists, if and where it can be accessed (e.g., Web address), and, if available, provide registration information including registration number.	NA
Eligibility criteria	6	Specify study characteristics (e.g., PICOS, length of follow-up) and report characteristics (e.g., years considered, language, publication status) used as criteria for eligibility, giving rationale.	4; Supplementary Table S6
Information sources	7	Describe all information sources (e.g., databases with dates of coverage, contact with study authors to identify additional studies) in the search and date last searched.	8; Figure 2
Search	8	Present full electronic search strategy for at least one database, including any limits used, such that it could be repeated.	4; Supplementary Table S5
Study selection	9	State the process for selecting studies (i.e., screening, eligibility, included in systematic review, and, if applicable, included in the meta-analysis).	4; Figure 2 and Supplementary Table S6
Data collection	10	Describe method of data extraction from reports (e.g., piloted forms, independently, in duplicate)	5

process	and any processes for obtaining and confirming data from investigators.		

Supplementary Table S4. (Continued)

Data items	11	List and define all variables for which data were sought (e.g., PICOS, funding sources) and any assumptions and simplifications made.	NA
Risk of bias in individual studies	12	Describe methods used for assessing risk of bias of individual studies (including specification of whether this was done at the study or outcome level), and how this information is to be used in any data synthesis.	NA
Synthesis of results	14	Describe the methods of handling data and combining results of studies, if done, including measures of consistency (e.g., I^2) for each meta-analysis.	5
Risk of bias across studies	15	Specify any assessment of risk of bias that may affect the cumulative evidence (e.g., publication bias, selective reporting within studies).	5
Additional analyses	16	Describe methods of additional analyses (e.g., sensitivity or subgroup analyses, meta-regression), if done, indicating which were pre-specified.	5
**RESULTS**			
Study selection	17	Give numbers of studies screened, assessed for eligibility, and included in the review, with reasons for exclusions at each stage, ideally with a flow diagram.	8
Study characteristics	18	For each study, present characteristics for which data were extracted (e.g., study size, PICOS, follow-up period) and provide the citations.	8; Table 4
Risk of bias within studies	19	Present data on risk of bias of each study and, if available, any outcome level assessment (see item 12).	11; Supplementary Figure S3
Results of individual studies	20	For all outcomes considered (benefits or harms), present, for each study: (a) simple summary data for each intervention group (b) effect estimates and confidence intervals, ideally with a forest plot.	Figure 3,4, and Supplementary Figure S1
Synthesis of results	21	Present results of each meta-analysis done, including confidence intervals and measures of consistency.	9

Supplementary Table S4. (Continued)

Risk of bias across studies	22	Present results of any assessment of risk of bias across studies (see item 15).	11; Supplementary Figure S3
Additional analysis	23	Give results of additional analyses, if done (e.g., sensitivity or subgroup analyses, meta-regression).	11; Supplementary Table S7, Figure 3B, and Supplementary Figure S2
**DISCUSSION**			
Summary of evidence	24	Summarize the main findings including the strength of evidence for each main outcome; consider their relevance to key groups (e.g., healthcare providers, users, and policy makers).	13
Limitations	25	Discuss limitations at study and outcome level (e.g., risk of bias), and at review-level (e.g., incomplete retrieval of identified research, reporting bias).	14-15
Conclusions	26	Provide a general interpretation of the results in the context of other evidence, and implications for future research.	15
**FUNDING**			
Funding	27	Describe sources of funding for the systematic review and other support (e.g., supply of data); role of funders for the systematic review.	15

Abbreviations: NA, not applicable.

Supplementary Table S5. Search strategy for associations of sex steroids and SHBG with NAFLD.

Data searched	via	Records	Records after duplicates removed
Embase	Embase.com	1606	1591
Medline ALL	Ovid	1115	436
Web of Science Core Collection	Web of Knowledge	579	249
Cochrane Central Register of Controlled Trials	Wiley	158	109
Other sources: Google Scholar (200 top ranked)		200	74
**Total**		**3658**	**2459**

Embase.com

('sex hormone'/de OR androgen/de OR estrogen/de OR 'androgen blood level'/de OR 'androgen deficiency'/de OR testosterone/de OR 'testosterone blood level'/de OR 'sex hormone binding globulin'/de OR estradiol/de OR 'estradiol blood level'/de OR hypogonadism/exp OR 'gonad dysfunction'/de OR 'ovary insufficiency'/exp OR 'testis function'/de OR 'hyperandrogenism'/exp OR prasterone/de OR 'prasterone sulfate'/de OR (((sex OR sexual OR gonad* OR testicular* OR ovar* OR reproductive) NEXT/3 hormone*) OR estrogen* OR oestrogen* OR androgen* OR hyperandrogen* OR hypoandrogen* OR testosterone* OR estradiol* OR oestradiol* OR hypogonad* OR hypergonad* OR ((gonad OR testis OR testes OR testicular OR ovar*) NEAR/3 (dysfunction* OR insufficien* OR failure* OR hypofunct* OR function*)) OR prasteron* OR dehydro-epiandrosteron* OR dehydroepiandrosteron*):ab,ti,kw) AND ('fatty liver'/exp OR 'liver fibrosis'/de OR (((fat* OR steato*) NEAR/3 (liver* OR hepat*)) OR steatohepat* OR ((liver OR hepat*) NEAR/3 (fibros* OR fibrot*)) OR nafld OR nafl OR nash):ab,ti,kw) NOT ([Conference Abstract]/lim OR [Note]/lim OR [Editorial]/lim) AND [english]/lim NOT ([animals]/lim NOT [humans]/lim)

Medline Ovid

("Gonadal Steroid Hormones"/ OR androgens/ OR testosterone/ OR "Sex Hormone-Binding Globulin"/ OR estradiol/ OR hypogonadism/ OR "hyperandrogenism"/ OR Dehydroepiandrosterone / OR Dehydroepiandrosterone Sulfate / OR (((sex OR sexual OR gonad* OR testicular* OR ovar* OR reproductive) ADJ3 hormone*) OR estrogen* OR oestrogen* OR androgen* OR hyperandrogen* OR hypoandrogen* OR testosterone* OR estradiol* OR oestradiol* OR hypogonad* OR hypergonad* OR ((gonad OR testis OR testes OR testicular OR ovar*) ADJ3 (dysfunction* OR insufficien* OR failure* OR hypofunct* OR function*)) OR prasteron* OR dehydro-epiandrosteron* OR dehydroepiandrosteron*).ab,ti,kw.) AND (exp Fatty Liver/ or Liver Cirrhosis/ OR (((fat* OR steato*) ADJ3 (liver* OR hepat*)) OR steatohepat* OR ((liver OR hepat*) ADJ3 (fibros* OR fibrot*)) OR nafld OR nafl OR nash).ab,ti,kw.) NOT (news OR comment OR editorial OR congresses OR abstracts).pt. AND english.la. NOT (exp animals/ NOT humans/)

Cochrane CENTRAL

((((sex OR sexual OR gonad* OR testicular* OR ovar* OR reproductive) NEXT/3 hormone*) OR estrogen* OR oestrogen* OR androgen* OR hyperandrogen* OR hypoandrogen* OR testosterone* OR estradiol* OR oestradiol* OR hypogonad* OR hypergonad* OR ((gonad OR testis OR testes OR

testicular OR ovar*) NEAR/3 (dysfunction* OR insufficien* OR failure* OR hypofunct* OR function*)) OR prasteron* OR dehydro-epiandrosteron* OR dehydroepiandrosteron*):ab,ti,kw) AND ((((fat* OR steato*) NEAR/3 (liver* OR hepat*)) OR steatohepat* OR ((liver OR hepat*) NEAR/3 (fibros* OR fibrot*)) OR nafld OR nafl OR nash):ab,ti,kw)

Web of science

TS=(((((sex OR sexual OR gonad* OR testicular* OR ovar* OR reproductive) NEAR/2 hormone*) OR estrogen* OR oestrogen* OR androgen* OR hyperandrogen* OR hypoandrogen* OR testosterone* OR estradiol* OR oestradiol* OR hypogonad* OR hypergonad* OR ((gonad OR testis OR testes OR testicular OR ovar*) NEAR/2 (dysfunction* OR insufficien* OR failure* OR hypofunct* OR function*)))) AND ((((nonalcohol* OR non-alcohol*) NEAR/2 (fat* OR steato*) NEAR/2 (liver* OR hepat*)) OR ((nonalcohol* OR non-alcohol*) NEAR/2 steatohepat*) OR ((liver OR hepat*) NEAR/2 (fibros* OR fibrot*)) OR nafld OR nafl OR nash))) AND DT=(article) AND LA=(english)

Google scholar

"sex|gonadal|testicular|ovarian hormone|hormones"|androgen|hyperandrogenism|hypoandrogenism|testosterone|estradiol|hypogonadism "non alcoholic fatty|steatohepatitis|hepatosteatosis"|"nonalcoholic fatty|steatohepatitis|hepatosteatosis"|nafl|nafld|nash

'sex|gonadal|testicular|ovarian hormone|hormones'|androgen|hyperandrogenism|hypoandrogenism|testosterone|estradiol|hypogonadism 'non alcoholic fatty|steatohepatitis|hepatosteatosis'|'nonalcoholic fatty|steatohepatitis|hepatosteatosis'|nafl|nafld|nash

Supplementary Table S6. Selection criteria used in the current systematic review and meta-analysis.

**Inclusion criteria**
1. Include cohort, cross-sectional, case-control and nested case-control studies.
2. Include studies which described:
(1) An association between steroids sex hormones [total estradiol , total testosterone, bioavailable estradiol, bioavailable testosterone, dehydroepianhdrosterone (DHEA) or dehydroepianhdrosterone sulfate (DHEAS)] and NAFLD (non-alcoholic fatty liver disease).
(2) An association between sex hormone-binding globulin (SHBG) and NAFLD (non-alcoholic fatty liver disease).
3. Include studies conducted in humans, all participants aged 18 years or older.
4. Only English language and no date restriction.
Exclusion criteria
1. Exclude abstracts, cost effectiveness studies, letters to the editor, conference proceedings, systematic reviews or meta-analyses.
2. Exclude studies conducted in animals.

Supplementary Table S7. Subgroup analysis of included studies for total testosterone with NAFLD.

Men	Studies	Participants	OR (95%CI)	P-value	I^2
**Overall population**	12	11055	0.59 (0.42-0.76)	<0.01	93%
**By continents**					
**Asia**	7	3840	0.57 (0.28-0.85)	<0.01	95.2%
**North America**	4	6460	0.59 (0.33-0.85)	0.31	16%
**Europe**	1	755	0.72 (0.39-1.05)	-	-
**By diagnostic methods**					
**Ultrasound**	8	5366	0.61 (0.39-0.84)	<0.01	94%
**Liver biopsy**	1	148	0.25 (-0.08-0.58)	-	-
**Liver enzymes**	1	2352	0.63 (0.47-0.79)	-	-
**CT**	1	837	0.98 (0.41-1.55)	-	-
**Fatty liver index**		2352	0.42 (0.11-0.72)	-	-
**By study size**					
**>1000**	3	6648	0.51 (0.22-0.80)	0.14	48.8%
**<1000**	9	4407	0.63 (0.40-0.86)	<0.01	89.2%
**By study design**					
**Cross-sectional**	8	9562	0.62 (0.41-0.83)	<0.01	94.5%
**Case-control**	2	308	0.26 (0.02-0.5)	0.90	0.0%
**Cohort**	2	1185	0.71 (-1.79-3.21)	0.23	31.9%

Abbreviations: NAFLD, non-alcoholic fatty liver disease; CT, computed tomography.

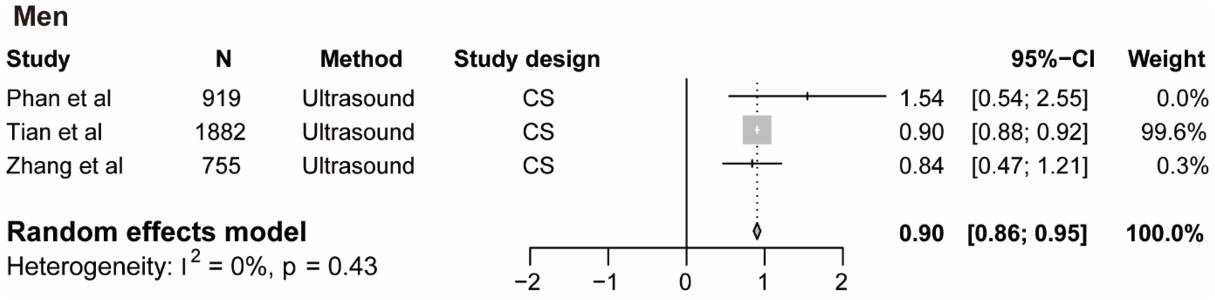

Supplementary Figure S1. Association of estradiol with NAFLD.

Abbreviations: NAFLD, non-alcoholic fatty liver disease.

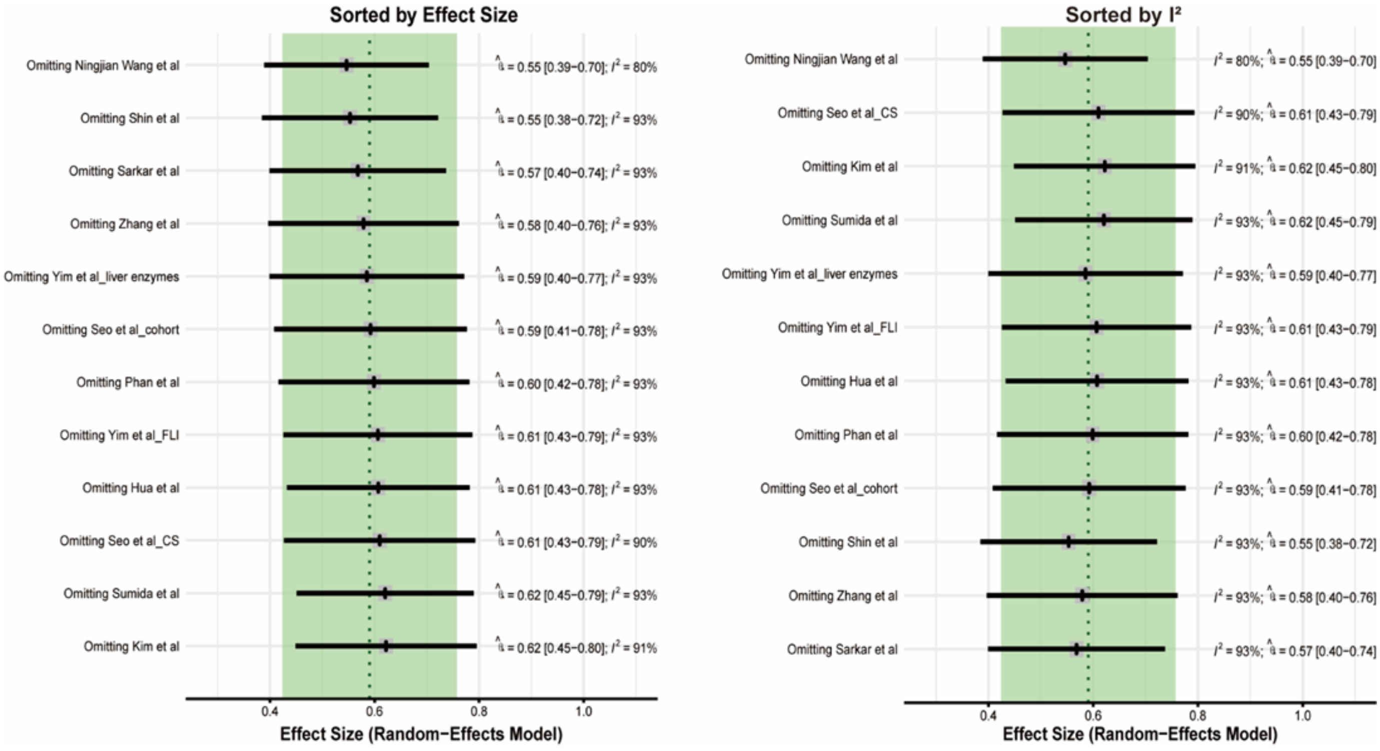

Supplementary Figure S2. Leave-one-out sensitivity analysis for the meta-analysis of testosterone and NAFLD among men. The left plot is sorted by effect size and the right plot is sorted by the heterogeneity I^2.

Abbreviations: NAFLD, non-alcoholic fatty liver disease; FLI, fatty liver index; CS, cross-sectional.

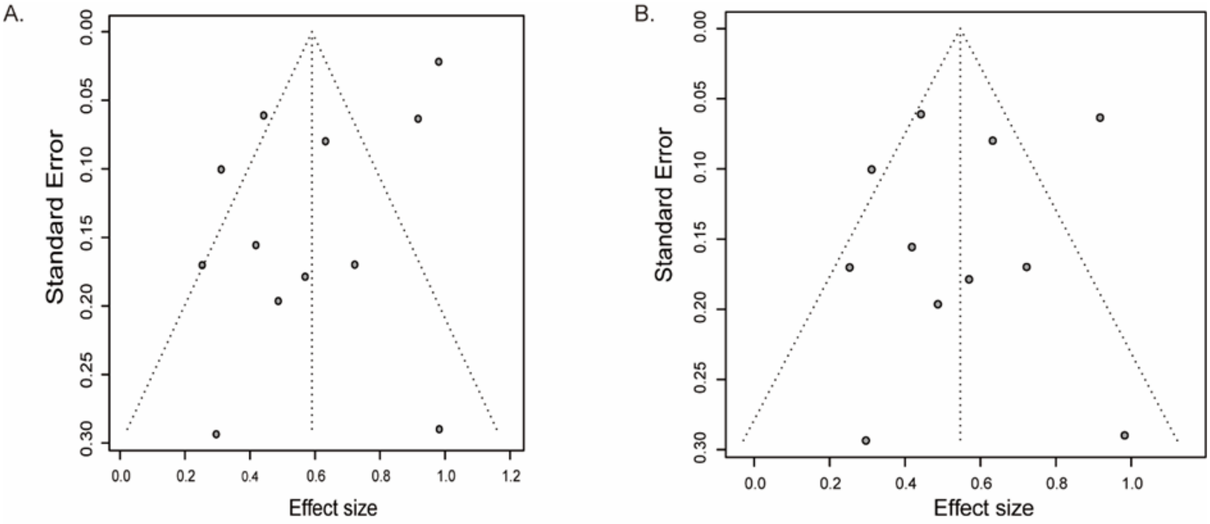

Supplementary Figure S3. Funnel plots and Egger's test in studies of total testosterone and NAFLD. The dotted lines show 95% confidence intervals around the overall summary estimate calculated using a random effect model; the p-value calculated using Egger's test were (A) 0.011 for all included studies on total testosterone among men and (B) 0.51 for excluded one study on total testosterone among men. Abbreviations: NAFLD, non-alcoholic fatty liver disease.
